# Amperometric Biosensor of Matrix Metalloproteinase-7 Enhanced by Pd-Functionalized Carbon Nanocomposites

**DOI:** 10.1186/s11671-018-2793-x

**Published:** 2018-11-22

**Authors:** Zheng Wei, Huiqiang Wang, Zhanfang Ma, Hongliang Han

**Affiliations:** Department of Chemistry, Capital Normal University, Beijing, 100048 China

**Keywords:** Matrix metalloproteinase-7, Tumour marker, Peptide cleavage, Impedance enhancer, Amperometric biosensor

## Abstract

Matrix metalloproteinase-7 plays a pivotal role in tumour progression and metastasis as an enzyme that can degrade the cell-matrix composition and cleave peptides between alanine and leucine in various biomolecular activation processes. In this work, a Pd-functionalised carbon nanocomposite was designed as a new impedance enhancer for an amperometric sensor of MMP-7. Pd nanoparticles in the enhancer can catalyse the oxidation of 4-chloro-1-naphthol with H_2_O_2_ to generate insoluble precipitation in situ, forming high-resistance precipitation on electrodes. In addition, poorly conductive carbon nanospheres of the nanocomposite increased the precipitation resistance, further causing a dramatic increase in resistivity of the enhancer and, subsequently, a significant decrease in current. This can significantly promote the current signal difference between the biosensor treated with and without the target analyte, which is directly related to the sensitivity of the amperometric biosensor. Overall, electrochemical biosensor can sensitively detect MMP-7 in the range of 100 fg mL^−1^ to 100 ng mL^−1^ with a limit of detection for MMP-7 of 17.38 fg mL^−1^.

## Background

Matrix metalloproteinase-7 (MMP-7), an enzyme that can degrade the composition of extracellular matrix [1], is highlighted for its pivotal role in tumour progression and metastasis [2, 3]. The content of MMP-7 in serum samples is associated with lymph node metastasis in patients with some cancers, such as salivary gland cancer [4], colon adenocarcinoma [5] and high-grade renal cell carcinoma [6]. Due to its various roles in physiological processes, high sensitive and accurate detection of MMP-7 has attracted intensive research attention [7], leading to the development of several approaches, including colorimetry [8], electrochemiluminescence (ECL) [9] and fluorescence resonance energy transfer (FRET) analysis [10]. Nevertheless, the limit of detection (LOD) of these approaches is typically in the picogram range and, thus, not low enough. Compared to these methods, electrochemical biosensors offer much more advanced MMP-7 detection capacities with lower LOD in femtogram level [11]. Moreover, some analytic methods have been constructed to meet the urgent requirement for ultrasensitive electrochemical detection of MMP-7 using electrochemical assays due to their low cost and miniaturisation [12].

In many electrochemical protocols, enzymatic biocatalytic reaction is applicable in signal amplification to promote the performance of the amperometric biosensor [13, 14]. However, it was well known that enzyme, the most widely used catalyst in catalytic reactions, had obvious shortcomings in both environmental-sensitive activity and low stabilities [15–19]. Therefore, developing a high efficient and stable catalyst is a top priority in constructing an ultrasensitive electrochemical assay for MMP-7 detection. Pd is a noble metal material with superior catalytic properties and possesses high chemical stability in catalytic reactions [20, 21]. In addition, carbon material can act as a chemical inert support in catalysts to adsorb noble metal materials and retain catalytic properties [22, 23].

Considering above situations, we designed a Pd-functionalised carbon nanocomposite as an impedance enhancer to dramatically increase the sensitivity of an amperometric assay of MMP-7, which has the following two functions. (1) Carbon nanospheres are a poor-conductive material [24]; (2) Pd nanoparticles can catalyse the oxidation of 4-chloro-1-naphthol with H_2_O_2_ to generate insoluble precipitation in situ, forming high-resistance precipitation on electrodes [25]. These two factors increase resistivity and significantly reduce current, which can remarkably improve the sensitivity of the biosensor to have a low LOD of 17.38 fg mL^−1^. The constructed Pd-functionalized nanocomposite for catalytic precipitation reaction is practical in amperometric assays of MMP-7 with high selectivity and sensitivity.

## Methods

### Material

HAuCl_4_·3H_2_O, H_2_PtCl_4_, 4-CN, glucose, H_2_O_2_ (30%), thrombin of bovine serum (TBS) were purchased from Alfa Aesar (Tianjin, China). Graphene oxide (GO) was purchased from JCNANO (Nanjing, China). Bovine serum albumin (BSA, standard grade) was commercially achieved from Beijing Xinjingke Biotechnologies Co., Ltd. (Beijing, China). The peptide (NH_2_-KKKRPLALWRSCCC-SH) was obtained from Science Peptide Biological Technology Co., Ltd. (Shanghai, China). Neuron-specific enolase (NSE) and prostate-specific antigen (PSA) were purchased from Shanghai Linc-Bio Science Co. Ltd. (Shanghai). Matrix metalloproteinase-2 (MMP-2) was purchased from Yeasen Biotechnologies Co., Ltd. (Shanghai, China). MMP-7 was provided from Sino Biological Inc. (Beijing, China). Clinical human serum samples were purchased by ZhongKe ChenYu Biotech (Beijing, China). All aqueous solutions were prepared with ultrapure water (resistivity > 18 MΩ cm). The phosphate buffer solution (PBS) contains 0.1 M KCl and 10 mM phosphate buffer (pH = 7.4).

### Apparatus

The microwave synthesis was carried out through CEM Discover® SP Microwave reactor (CEM, USA). Scanning electron microscope (SEM) images were obtained by using HITACHI S-4800 SEM (HITACHI, Japan). Transmission electron microscope (TEM) images were obtained on HITACHI H7650 TEM (HITACHI, Japan). Energy dispersive X-ray spectroscopy (EDS) was determined on HITACHI SU8010 SEM (HITACHI, Japan). All electrochemical measurements were carried out on CHI600 electrochemical workstation (Chenhua Instruments Co., Shanghai, China). A glassy carbon electrode (GCE) (4 mm in diameter) was used as working electrode, a platinum wire and a Ag/AgCl electrode were as counter electrode and reference electrode, thus a three electrochemical system in experiment was constructed. High-resolution transmission electron microscope (HR-TEM) images were performed by Tecnai G^2^F30 TEM under 300 kV accelerating.

### Synthesis of the Pd-Functionalized Carbon Nanocomposites

Pd-functionalized carbon nanocomposites (Pd-CNCs) were synthesised according to a previously reported literature [26]. Briefly, 4 g of glucose was dissolved into 40 mL of ultrapure water to form a clear solution. The above solution was then transferred into a 50 mL Teflon-lined stainless-steel autoclave and kept at 170 °C for 5 h. After reaction, the transparent solution turned to dark brown. The obtained carbon nanospheres were collected via centrifugation and washed by ultrapure water/ethanol for several times. The resulted products were redisposed in 8 mL of ultrapure water.

To functionalize Pd nanoparticles on carbon nanospheres [24], 1 mL carbon nanospheres suspension was mixed with 25 μL HPdCl_4_ solution. The mixture was reacted in microwave reaction instrument (250 W) at 100 °C for 15 min and then cooled down to the room temperature naturally. Next, the obtained Pd-carbon nanospheres were centrifuged, washed by ultrapure water and redisposed in 1 mL of ultrapure water.

To avoid the unspecific adsorption of MMP-7, BSA was further modified on the Pd-carbon nanospheres. The nanospheres suspension was stirred with 100 μL BSA solution (1 wt%) for 1 h under room temperature. After several centrifugation and washing steps, the BSA-modified Pd-carbon nanospheres were redisposed in ultrapure water and stored in 4 °C for further experiments.

### Electrodeposition Au-rGO on GCE

Before modification, the GCE was polished with 0.05 μm alumina slurry and sonication washed in ultrapure water and ethanol, respectively. The electrodepositing solution was configured by the following steps [27]. Firstly, 8 mg GO powder was dispersed in 20 mL ultrapure water under sonication for 2 h. Then, 200 μL HAuCl_4_ solution (4 wt%) was added into the GO suspension Subsequently, the electrodeposition of Au-rGO on GCE by cyclic voltammetry (CV) technique in the range between 1.5 and − 1.5 V with the scan rate of 50 mV s^−1^ in the above electrodepositing solution. Finally, the Au-rGO deposited GCE (Au-rGO/GCE) was washed by ultrapure water to remove the residual electrodepositing solution and then blow dried by nitrogen at room temperature.

### Fabrication of the Biosensor

The Au-rGO/GCE was incubated with 40 μL peptide solution (50 μM) for 40 min at 37 °C. Subsequently, the modified peptide on the GCE was activated with 50 μL glutaraldehyde solution (0.10 wt%) for another 30 min (peptides/Au-rGO/GCE). Then, 20 μL Pd-CNCs suspension was dropped on the peptide-modified electrode for 1 h (Pd-CNCs/peptides/Au-rGO/GCE). After each modification step, the modified electrode was washed by ultrapure water.

### Electrochemical Measurement

Eighty microliters of MMP-7 solution (1 ng mL^−1^) was incubated with the Pd-CNCs/peptides/Au-rGO/GCE for 1 h at 37 °C and sufficiently washed by ultrapure water. Then, 50 μL of 1.0 mM 4-CN solution containing 10 mM H_2_O_2_ was dropped to proceed precipitation reaction for 50 min. Finally, the square wave voltammetry (SWV) measurement was conducted from − 0.2 to 0.6 V in 5 mM [Fe(CN)_6_]^3−/4−^ phosphate-buffered solution (0.1 M, pH = 7.4) with pulse amplitude of 25 mV and an increase potential of 4 mV s^−1^.

## Results and Discussion

### Principle of the Peptide Cleavage Biosensor

**Scheme 1:**
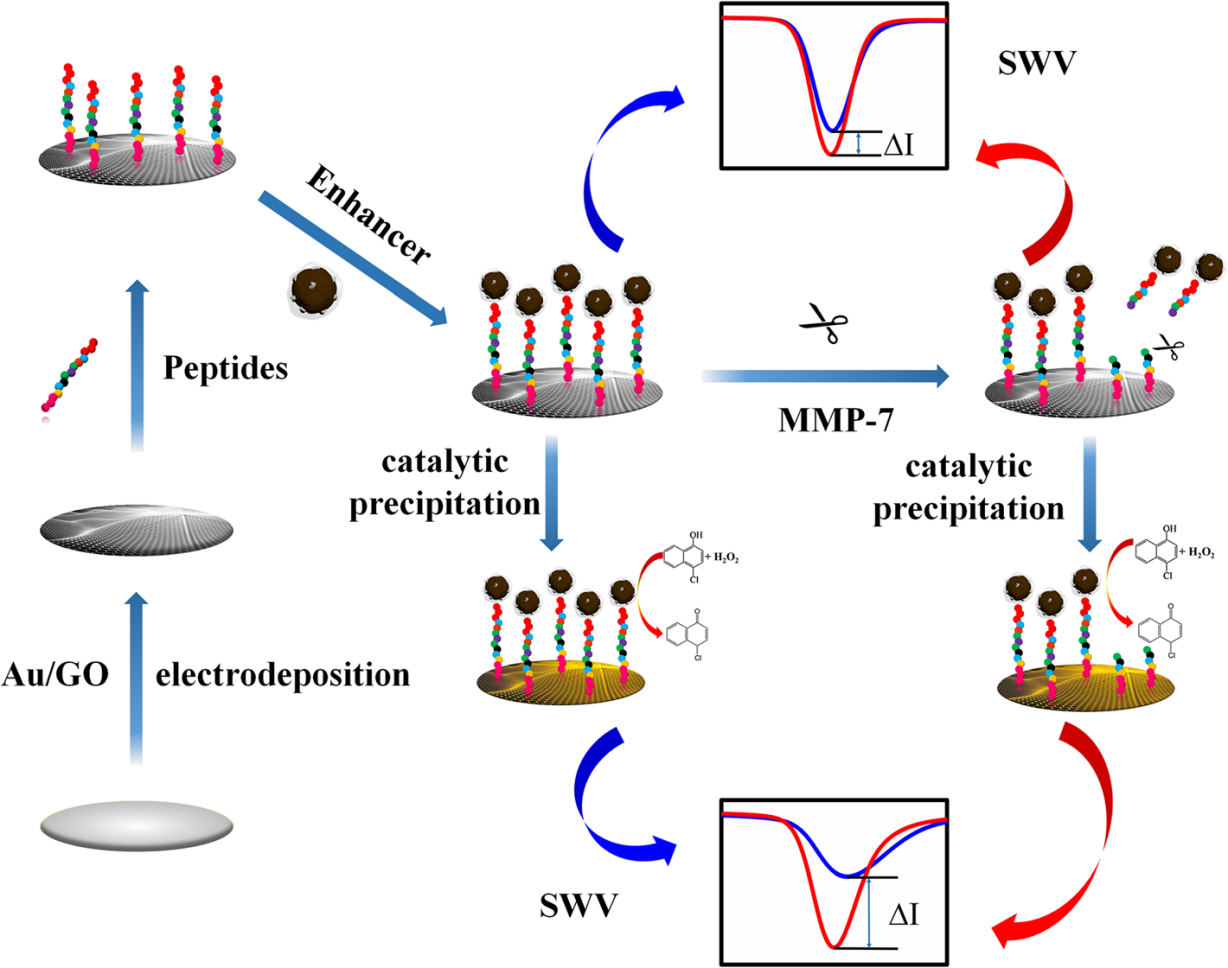
Schematic illustration of the Pd-functionalized carbon nanosphere as an impedance enhancer for amperometric assay of MMP-7

The construction process of the biosensor based on peptide cleavage is illustrated in Scheme 1. First, Au-rGO was deposited on the glassy carbon electrode (GCE) through an electrochemical method, then peptides were immobilised on Au-rGO through Au-S bonding. Pd-CNCs were composed as a catalytic enhancer, and glutaraldehyde was selected as the chemical linker between the peptide and enhancers. 4-CN and H_2_O_2_ were employed as catalysed precipitation substrates to enhance impedance. Particularly, MMP-7 was chosen as the peptide-cleavage enzyme between alanine (A) and leucine (L) in peptides [8], which caused an indistinctive current signal change measured by SWV. Electrodeposition of Au-rGO on the GCE has two advantages: (1) Au-rGO can effectively improve the conductivity of the sensing interface; and (2) it allows the sites to immobilise peptides. Taking advantage of the high impedance and catalytic performance of the Pd-CNCs, ΔI was then amplified by replacing the Pd-CNCs with the specific peptide (NH2-KKKRPLALWRSCCC-SH) cleavage. This phenomenon is contributed to the poor electrical conductivity of Pd-CNCs and high impedance of precipitation generated from the oxidation of 4-CN with H_2_O_2_ catalysed by Pd-CNCs.

**Fig. 1:**
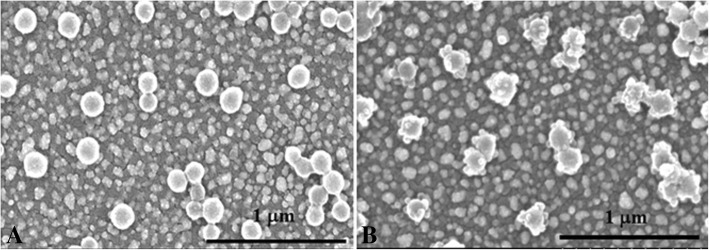
SEM images of Pd-CNCs/peptides/Au-rGO/GCE (**a**) treated with catalytic precipitation reaction (**b**)

The catalytic precipitation reaction on the GCE was further characterised by scanning electron microscopy (SEM) by incubating Pd-CNCs on the surface of the peptides/Au-rGO/GCE (Fig. 1a). Pd-CNCs were coated with an insoluble layer after the precipitation reaction, indicating that the insoluble poor-conductivity layer was formed on the modified electrode (Fig. 1b). Hence, a biosensor for ultrasensitive detection of MMP-7 was successfully established from the sensitivity amplification attracted by both peptide cleavage and catalytic precipitation.

### Characterisation of the Pd-CNCs

**Fig. 2:**
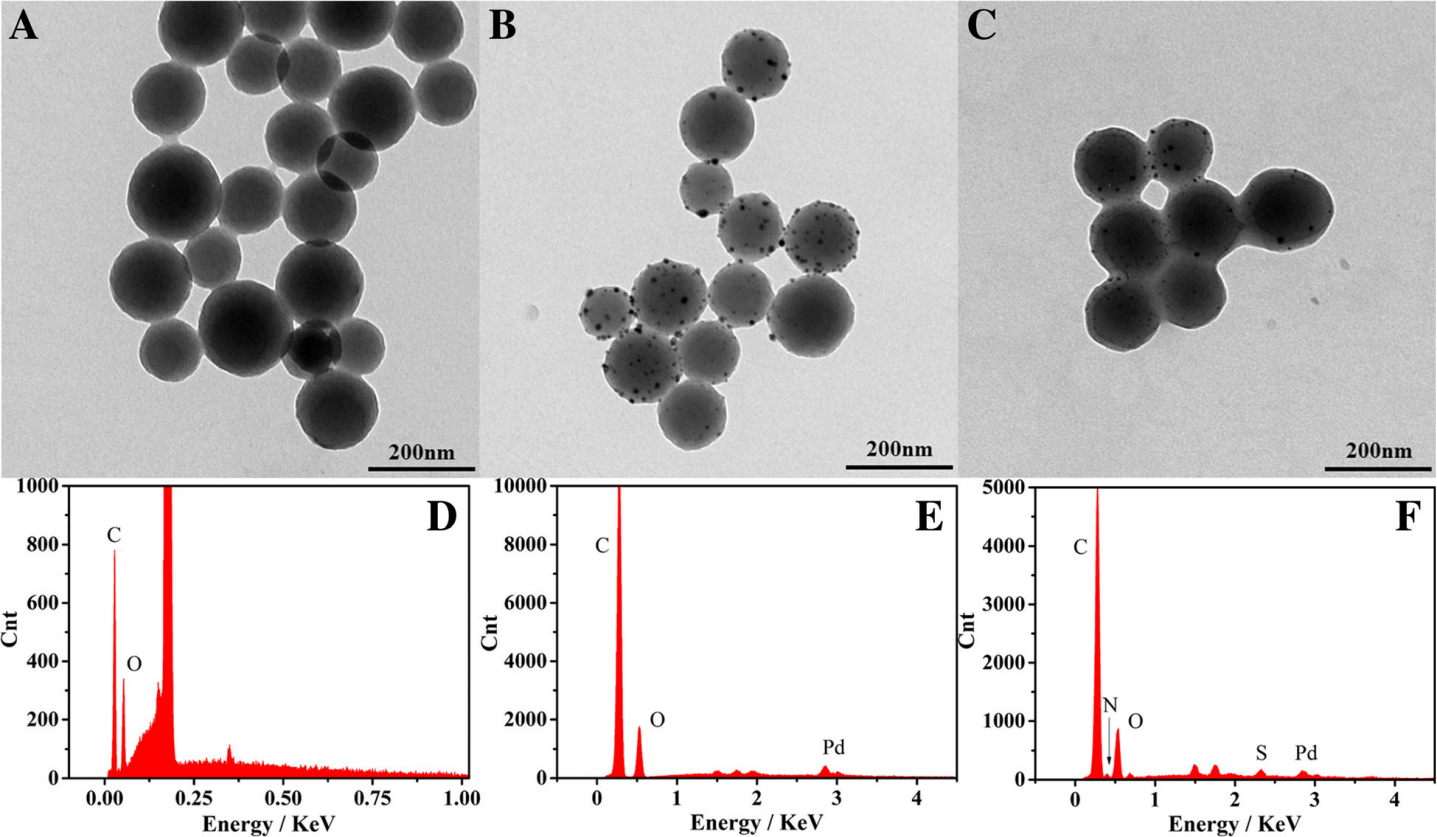
TEM micrographs of carbon nanosphere (**a**), Pd-carbon nanospheres (**b**) and Pd-CNCs (**c**) EDS of carbon nanosphere (**d**), Pd-carbon nanospheres (**e**) and Pd-CNCs (**f**)

**Fig. 3:**
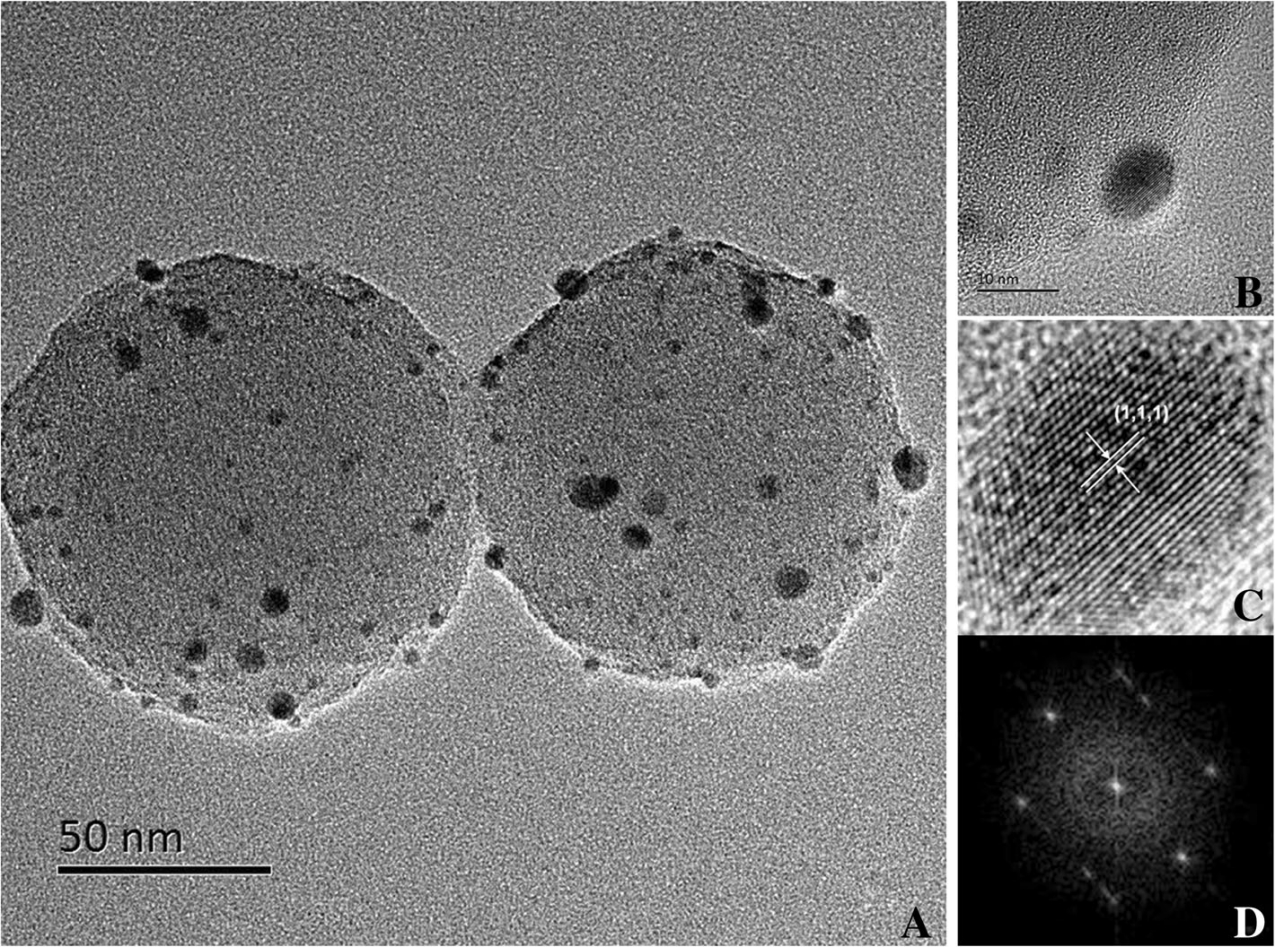
HR-TEM images of Pd-carbon nanospheres (**a**), magnified images of Pd nanoparticles (**b, c**) and FFT images of Pd nanoparticles (**d**)

By a typical hydrothermal method, homogeneous Pd-CNCs were successfully prepared. The morphologies of carbon nanospheres, Pd-carbon nanospheres and Pd-CNCs were characterised by transmission electron microscopy (TEM) and energy dispersive X-ray spectrometry (EDS). Pd nanoparticles were formed and distributed on the carbon nanospheres following the microwave reaction (Fig. 2b), demonstrating the successful preparation of Pd-carbon nanospheres with diameters of 150 nm (Fig. 2a). The morphologies of Pd-CNCs are shown in Fig. 2c. The EDS results confirm the chemical compositions of carbon nanoparticles, Pd-carbon nanospheres and Pd-CNCs, which shows the carbon nanoparticles contain C and O (Fig. 2d). After the functionalization of Pd nanoparticles, Pd also existed in the spectra of Pd-carbon nanospheres (Fig. 2e), while S and N found in the spectra of Pd-CNCs originate from BSA, which was used to block Pd-carbon nanospheres (Fig. 2f). These results intuitively reveal the successful synthesis of the Pd-CNC catalytic enhancers. HR-TEM images of Pd-carbon nanospheres (Fig. 3a) illustrate that the functionalised Pd nanoparticles (Fig. 3b) are in face-center cubic (FCC) phase with an observable (1,1,1) lattice plane (Fig. 3c). Corresponding fast Fourier transformation (FFT) images (Fig. 3d) also support the FCC crystalline nature of Pd nanoparticles.

### Characterisation of Construction Procedures of the Biosensor

**Fig. 4:**
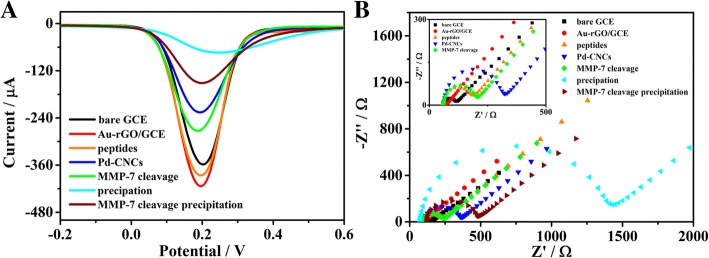
SWV (**a**) and EIS (**b**) responses of modification process of electrode in 5 mM [Fe(CN)_6_]^3−/4-^ phosphate buffered (0.1 M, pH = 7.4)

The construction procedures of the biosensor were monitored by SWV measurements (Fig. 4a) and electrochemical impedance spectroscopy (EIS) (Fig. 4b). Compared to the current signal of bare GCE (curve bare GCE), the peak current in the potential range increased to about 420 μA (curve Au-rGO/GCE) after electrodeposition of Au-rGO, which may be attributed to the excellent conductivity of Au-rGO. Then, the signal current peak decreased after the immobilisation of peptides on the electrode (curve peptides) and was further reduced after the linkage between peptides and Pd-CNCs due to the high impedance of the peptides and enhancers (curve Pd-CNCs). After incubating with MMP-7, the peak current increased (curve MMP-7 cleavage), which may be caused by the specific cleavage of peptides by MMP-7 and partial removal of enhancers from the electrode surface. Under the same conditions, the current change (ΔI_1_) between curves “Pd-CNCs” and “MMP-7 cleavage” was calculated to be 48.7 μA. Then, the peak current was reduced after the catalytic precipitation reaction of 4-CN, with a current peak of 136.1 μA (curve MMP-7 cleavage precipitation). In contrast, the biosensor without MMP-7 induced a lower current peak (54.9 μA, curve precipitation), which was stated as the blank signal, after the catalysed precipitation reaction. In the circumstances, ΔI_2_ was the current signal difference between curves “precipitation” and curve “MMP-7 cleavage precipitation”, increasing from 48.7 to 81.2 μA. EIS was also used to monitor the fabrication of the biosensor. The electron transfer resistance corresponded to the semicircle diameter of the Nyquist plots (Fig. 4b). For comparison, the plot of the peptides/Au-rGO/GCE (curve peptides) displays a larger semicircle, and thus larger resistance, than those of Au-rGO/GCE (curve Au-rGO/GCE) and bare GCE (curve bare GC) which has the smallest circle, indicating that peptides were successfully modified on the GCE. The resistance increased when peptides were linked with the enhancer via glutaraldehyde (curve Pd-CNCs). The diameter of semicircle slightly decreased (curve MMP-7 cleavage) after incubation with MMP-7, which may be ascribed to the MMP-7 specifically cleaved the peptides. In contrast, the resistance significantly increased when the biosensor was dipped in the solution of 4-CN and H_2_O_2_ (curve precipitation)_._ Undergoing both peptide-cleavage and catalytic precipitation reaction, the resistance decreased obviously (curve MMP-7 cleavage precipitation).

### Optimization of Detection Conditions

**Fig. 5:**
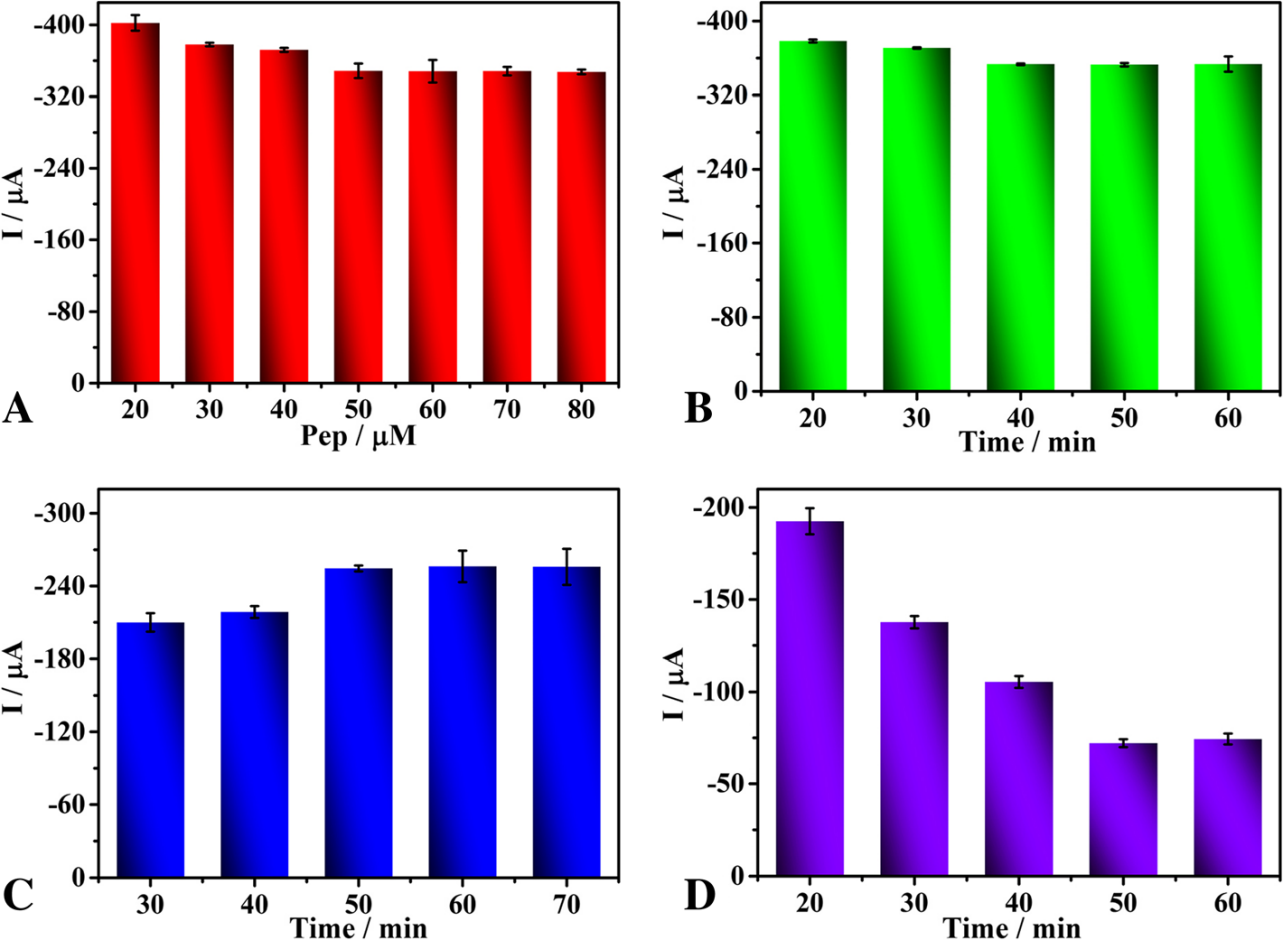
Effects of peptide concentration (**a**), incubation time of 50 μM peptide (**b**), peptide cleavage time (**c**) and precipitation reaction time (**d**) on the current responses of the biosensor

The quantity and incubation time of peptides, as important factors for the detection performance of the biosensor, were further optimised. It was found that the current signal decreased accordingly when the peptide concentration increased from 20 to 40 μM and kept constant between 50 and 80 μM (Fig. 5a). To avoid nonspecific adsorption, we chose 50 μM for subsequent measurements and then optimised the incubation time of the 50 μM peptide (Fig. 5b). The SWV current decreased gradually from 20 to 40 min, then remained constant after 40 min. Thus, 40 min was chosen as the incubation time for peptides. The cleavage reaction time is also a significant factor for the analytical performance of the biosensor (Fig. 5c). The signal of the biosensor increased when the cleavage time ranged between 30 and 50 min, then remained constant after 50 min, suggesting that the peptide was completely cleaved. Thus, 50 min was chosen as the cleavage time for the following experiments. The precipitation reaction, which also influences the sensitivity of the biosensor (Fig. 5d), was found to increase within 50 min, and the electrochemical signal decreased continuously. Since the current signal maintained constant with a further increase in reaction time, 50 min was selected as the precipitation reaction time for the following assay.

### Performance of Proposed Biosensor

**Fig. 6:**
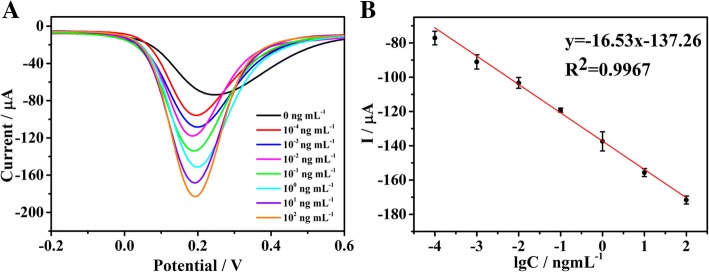
**a** SWV responses of electrochemical detection for MMP-7 in 5 mM [Fe(CN)_6_]^3−/4−^ phosphate buffered (0.1 M, pH = 7.4) at the concentrations from 100 fg mL^−1^ to 100 ng mL^−1^. **b** Linear calibration curve of current and the logarithm of the concentration of MMP-7. The error bars are standard deviations for *n* = 3

After incubation with different concentrations of MMP-7 under the optimal conditions, the performance of the proposed biosensor was determined. As shown in Fig. 6, with decreasing concentration of MMP-7, the SWV signal decreased. The calibration plot reveals a good linear relationship between the current peaks and the logarithm of the analyte concentrations in the range from 0.1 pg mL^−1^ to 100 ng mL^−1^. The linear equation was determined to be (*I* = − 16.53lgCMMP-7-137.26) with a correlation coefficient of 0.9967. The detection limit of the biosensor was 17.38 fg mL^−1^ for MMP-7 at a signal-to-noise ratio of 3 (S/*N* = 3; is the standard deviation of the signal in a blank solution). Compared with recent reports on peptide-cleavage detection of MMP-7, the biosensor exhibited a better analytical performance (Table 1).

**Table 1:** Comparison of some recently reported biosensor for detection of MMP-7

Material and method	Detection limit	Detection range	Reference
DNA enzyme-decorated DNA nanoladders Differential pulse voltammetry	0.05 pg mL^−1^	2 × 10^− 4^–20 ng mL^−1^	Kou et al. 2016 [14]
Au/Ag@SiO_2_ Fluorescence resonance energy transfer	0.22 pg mL^−1^	1 × 10^− 2^–100 ng mL^−1^	Lei et al. 2017 [10]
Gold nanoparticles Colorimetry	0.1 μg mL^−1^	0–2 μg mL^−1^	Chen et al. 2013 [8]
Magnetic peptide−DNA probe Square wave voltammetry	0.02 pg mL^−1^	1 × 10^− 4^–50 ng mL^−1^	Wang et al. 2017 [28]
BSA-Pd-SAM-PDA probe Square wave voltammetry	3.1 fg mL^−1^	1 × 10^− 5^–10 ng mL^−1^	Zheng et al. 2018 [11]
Pd-CNCs enhancer Square wave voltammetry	17.38 fg mL^−1^	1 × 10^− 4^–100 ng mL^−1^	This work

### Evaluation of Performance of Immunosensor

**Fig. 7:**
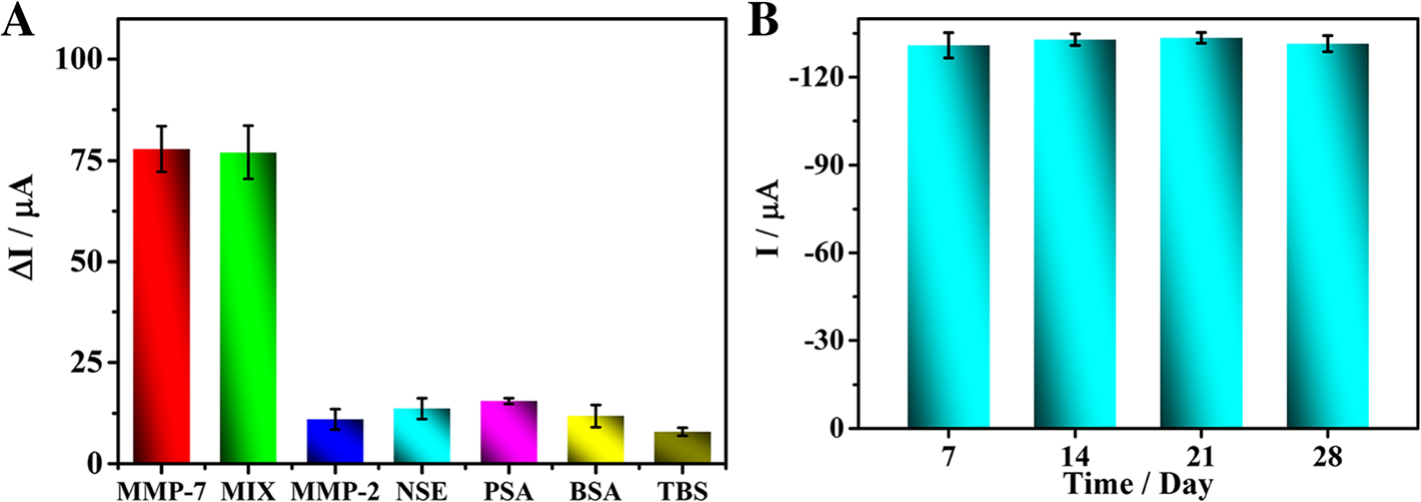
SWV current responses (**a**) about anti-interference ability of the biosensor (the error bars are standard deviations for *n* = 3). The concentrations of distractions: MMP-2 (100 ng mL^−1^), NSE (100 ng mL^−1^), PSA (100 ng mL^−1^), THR (100 ng mL^−1^) and BSA (100 ng mL^−1^), respectively. The mixture contains all those distractions (100 ng mL^−1^) and MMP-7 (1 ng mL^−1^). SWV responses (**b**) of the proposed biosensors stored at 4 °C for different time for the detection of 1 ng mL^−1^ MMP-7

To assess the reproducibility of the biosensor, three modified electrodes were incubated with 0.01, 0.1, 1, 10 and 100 ng mL^−1^ MMP-7. The corresponding standard deviations were calculated to be 1.3%, 1.4%, 4.0%, 1.0% and 3.0%, respectively, indicating good reproducibility of the biosensor. MMP-2, NSE, PSA, TBS and BSA were used as distractions to analyse the specificity of this biosensor. No obvious responses were observed when the biosensor was incubated with individual mixtures of 1 ng mL^−1^ MMP-7 with (100 ng mL^−1^ each) MMP-2, NSE, PSA, TBS and BSA. Compared to the biosensor incubated with only 1 ng mL^−1^ MMP-7, the current signals of each mixture were almost the same (Fig. 7a), which demonstrates that the biosensor displays excellent specificity for MMP-7. To investigate stability, the constructed biosensor was stored at 4 °C for 28 days, and its performance for MMP-7 detection was evaluated every 7 days (Fig. 7b). The relative standard deviations (RSD) between parallel experiments were all less than 10%, implying remarkable stability of the biosensor.

To investigate the practicability of the proposed method, recovery experiments were carried out. The recoveries ranged from 86.3 to 117.2% (Table 2), further indicating the promising practicability of the biosensor in clinical analyses.

**Table 2:** Recovery tests for MMP-7 in human serum samples

Added (ng mL^−1^)	Detected (ng mL^−1^)	Average recovery (%)
1	0.8627	86.27
1	1.172	117.2
0.1	0.1038	103.8
0.1	0.09958	99.58
0.01	0.01028	102.8
0.01	0.01000	100
0.001	0.001089	108.9
0.001	0.000943	94.3

## Conclusions

In summary, a Pd-functionalized carbon nanocomposite was fabricated as a novel impedance enhancer, which reveals promising H_2_O_2_ catalytic performance for 4-CN oxidation. Utilising the high impedance of insoluble oxidised 4-CN precipitation on the electrode, an amperometric biosensor for detection of MMP-7 was constructed. The biosensor possesses comparable sensitivity, a broad detection range, good practicability and outstanding selectivity to the detection of MMP-7, which suggests it potential application in various bio-applications. Our work further highlights the importance of the impedance enhancer in improving the performance of amperometric assays, encouraging the fabrication of new enhancers with advanced catalytic activity and high resistance.

## Abbreviations

4-CN: 4-Chloro-1-naphthol
BSA: Bovine serum albumin
EDS: Energy dispersive X-ray spectroscopy
FCC: Face-center cubic
FFT: Fast Fourier transformation
GCE: Glassy carbon electrode
GO: Graphene oxide
HR-TEM: High-resolution transmission electron microscope
LOD: Limit of detection
MMP-2: Matrix metalloproteinase-2
MMP-7: Matrix metalloproteinase-7
NSE: Neuron specific enolase
PBS: Phosphate buffer solution
Pd-CNCs: Pd-functionalized carbon nanocomposites
PSA: Prostate-specific antigen
RSD: Relative standard deviations
SEM: Scanning electron microscope
SWV: Square wave voltammetry
TBS: Thrombin of bovine serum
TEM: Transmission electron microscope

## References

[1] Szarvas T, Becker M, vom Dorp F, Gethmann C, Totsch M, Bankfalvi A (2010). Matrix metalloproteinase-7 as a marker of metastasis and predictor of poor survival in bladder cancer. Cancer Sci.

[2] Miwa S, Miyagawa S, Soeda J, Kawasaki S (2002). Matrix metalloproteinase-7 expression and biologic aggressiveness of cholangiocellular carcinoma. Cancer.

[3] Tanioka Y, Yoshida T, Yagawa T, Saiki Y, Takeo S, Harada T (2003). Matrix metalloproteinase-7 and matrix metalloproteinase-9 are associated with unfavourable prognosis in superficial oesophageal cancer. Br J Cancer.

[4] Luukkaa H, Klemi P, Hirsimaki P, Vahlberg T, Kivisaari A, Kahari VM (2010). Matrix metalloproteinase (MMP)-7 in salivary gland cancer. Acta Oncol.

[5] Zucker S, Vacirca J (2004). Role of matrix metalloproteinases (MMPs) in colorectal cancer. CANCER METAST REV.

[6] Lu H, Yang Z, Zhang H, Gan M, Zhou T, Wang S (2013). The expression and clinical significance of matrix metalloproteinase 7 and tissue inhibitor of matrix metalloproteinases 2 in clear cell renal cell carcinoma. Exp Ther Med.

[7] Cheng W, Chen Y, Yan F, Ding L, Ding S, Ju H (2011). Ultrasensitive scanometric strategy for detection of matrix metalloproteinases using a histidine tagged peptide-Au nanoparticle probe. Chem Commun (Camb).

[8] Chen P, Selegard R, Aili D, Liedberg B (2013). Peptide functionalized gold nanoparticles for colorimetric detection of matrilysin (MMP-7) activity. Nanoscale.

[9] Gao H, Dang Q, Xia S, Zhao Y, Qi H, Gao Q (2017). Highly selective electrogenerated chemiluminescence biosensor for simultaneous detection of matrix metalloproteinase-2 and matrix metalloproteinase-7 in cell secretions. Sensors Actuators B Chem.

[10] Lei Z, Zhang H, Wang Y, Meng X, Wang Z (2017). Peptide microarray-based metal enhanced fluorescence assay for multiple profiling of matrix metalloproteinases activities. Anal Chem.

[11] Zheng Y, Ma Z (2018). Dual-reaction triggered sensitivity amplification for ultrasensitive peptide-cleavage based electrochemical detection of matrix metalloproteinase-7. Biosens Bioelectron.

[12] Tang Z, Ma Z (2017). Multiple functional strategies for amplifying sensitivity of amperometric immunoassay for tumor markers: a review. Biosens Bioelectron.

[13] Tang Z, Wang L, Ma Z (2017). Triple sensitivity amplification for ultrasensitive electrochemical detection of prostate specific antigen. Biosens Bioelectron.

[14] Kou BB, Zhang L, Xie H, Wang D, Yuan YL, Chai YQ (2016). DNA enzyme-decorated DNA Nanoladders as enhancer for peptide cleavage-based electrochemical biosensor. ACS Appl Mater Interfaces.

[15] Sun Z, Glebe U, Charan H, Boker A, Wu C (2018). Enzyme-polymer conjugates as robust Pickering interfacial biocatalysts for efficient biotransformations and one-pot Cascade reactions. Angew Chem Int Ed Engl.

[16] Wang X, Liu X, Yan X, Zhao P, Ding Y, Xu P (2011). Enzyme-nanoporous gold biocomposite: excellent biocatalyst with improved biocatalytic performance and stability. PLoS One.

[17] Cheng MB, Wang JC, Li YH, Liu XY, Zhang X, Chen DW (2008). Characterization of water-in-oil microemulsion for oral delivery of earthworm fibrinolytic enzyme. J Control Release.

[18] Chiu NF, Chen CC, Yang CD, Kao YS, Wu WR (2018). Enhanced Plasmonic Biosensors of hybrid gold nanoparticle-graphene oxide-based label-free immunoassay. Nanoscale Res Lett.

[19] He G, Tian L, Cai Y, Wu S, Su Y, Yan H (2018). Sensitive nonenzymatic electrochemical glucose detection based on hollow porous NiO. Nanoscale Res Lett.

[20] Stahl SS (2004). Palladium oxidase catalysis: selective oxidation of organic chemicals by direct dioxygen-coupled turnover. Angew Chem Int Ed Engl.

[21] Goodman ED, Dai S, Yang A-C, Wrasman CJ, Gallo A, Bare SR (2017). Uniform Pt/Pd bimetallic nanocrystals demonstrate platinum effect on palladium methane combustion activity and stability. ACS Catal.

[22] Xu F, Tang Z, Huang S, Chen L, Liang Y, Mai W (2015). Erratum: facile synthesis of ultrahigh-surface-area hollow carbon nanospheres for enhanced adsorption and energy storage. Nat Commun.

[23] Liao X, Ju H (2015). In situ quantitation of intracellular microRNA in the whole cell cycle with a functionalized carbon nanosphere probe. Chem Commun (Camb).

[24] Xu T, Liu N, Yuan J, Ma Z (2015). Triple tumor markers assay based on carbon-gold nanocomposite. Biosens Bioelectron.

[25] Tang Z, Han G-H, Jung E, M-g S, Lee K-Y, Kim W-S (2018). Synthesis of Cu-Pd nanoplates and their catalytic performance for H_2_O_2_ generation reaction. Mol Catal.

[26] Cui R, Liu C, Shen J, Gao D, Zhu J-J, Chen H-Y (2008). Gold nanoparticle-colloidal carbon nanosphere hybrid material: preparation, characterization, and application for an amplified electrochemical immunoassay. Adv Funct Mater.

[27] Chen Y, Liu X, Zhang S, Yang L, Liu M, Zhang Y (2017). Ultrasensitive and simultaneous detection of hydroquinone, catechol and resorcinol based on the electrochemical co-reduction prepared Au-Pd nanoflower/reduced graphene oxide nanocomposite. Electrochim Acta.

[28] Wang D, Chai Y, Yuan Y, Yuan R (2017). A peptide cleavage-based ultrasensitive electrochemical biosensor with an ingenious two-stage DNA template for highly efficient DNA exponential amplification. Anal Chem.

